# Direct light-induced propulsion of vessels filled with a suspension of graphene particles and methanol

**DOI:** 10.1038/s41598-020-59123-y

**Published:** 2020-02-10

**Authors:** W. Strek, P. Wiewiorski, W. Mista, R. Tomala

**Affiliations:** Institute of Low Temperature and Structure Research, Polish Academy of Science, Wroclaw, Poland

**Keywords:** Energy, Energy science and technology

## Abstract

The direct propulsion of glassy capsule filled with solution of methanol and disperse graphene foam (GF) particles under irradiation with infrared LED is reported. The vertical propulsion occurred after irradiation of transparent glassy bottom. The velocity of propulsion was dependent of light irradiation power. It was observed that with irradiation the GF particles moved violently and vertically with direction of lighting. It was found that upon light irradiation there is generated efficiently hydrogen upon solution surface. The mechanism of propulsion effect was discussed in terms of the explosive hydrogen-oxygen reaction.

## Introduction

Recently Zhang *et al*.^1^ have reported a laser-induced propulsion (LIP) of a bulk graphene sponge in vacuum. The horizontal and vertical propulsion or rotation of bulk graphene was dependent on laser light wavelength and laser power density. The observed phenomenon was discussed in terms of electron emission giving input to kinetic energy of graphene sponge. The authors^1^ have suggested the potential application of LIP effect for space transportation. As another mechanism for laser light propulsion on a basis of classical arguments was proposed by Lei Wu *et al*.^2^ in the correspondence to the Editor. Following the estimation of propulsion force provided by light–induced ejected electrons to be too small than opposite gravitational force they have pointed on the radiometric force of Reynolds^3^ to be responsible for the effect. The propulsion caused by radiometric force is due to the difference of temperature at the bottom and the top of laser illuminated graphene sponge bulk. In the reply to the correspondence by Wu *et al*. Zhang *et al*.^4^ have supplied additional strong arguments to emphasize the role of hot ejected electrons for laser induced movement of bulk graphene sponge. The phenomena of laser induced propulsion was a subject of many investigations^5–15^. A review of different nanoscale photo- and redox –driven artificial molecular motors was recently presented by Baroncini *et al*.^16^. The light driven levitation of graphene sponge was discussed by Gabor^6^. Laser initiated water vapor propulsion as drive for space vehicles was presented Minami and Uchida^8^. The discussion of application of physical mechanisms for photonic space propulsion was discussed by Levchenko *et al*.^9^. Propulsion of glass micro-spheres irradiated by nanosecond laser pulses were discussed by Yu *et al*.^11^. The studies of underwater laser propulsion of metallic target materials was reported Qiang *et al*.^10^. The propulsion mechanism for catalytic microjet engines used in microrobotics was proposed by Fomin *et al*.^17^. Application of laser-driven propulsion of graphene flakes for microjet engines was investigated by Chengbin Qin *et al*.^18^.

In present work we report the observation of light-induced propulsion of massive glass vessel (capsule) filled with suspension of methanol with dispersed fine graphene foam (GF) particles. The capsule was illuminated with CW infrared light emitting diode (LED) radiation. The strength of propulsion force was dependent of excitation power density. It was measured to be over 20 N and exceeded 10^5^ times the gravity force of capsule. The mechanism of the propulsion effect was discussed in terms of photon induced generation of hydrogen following photoelectron emission from graphene particles. This mechanism may be applied for transportation of large scale vehicles.

## Experimental

**Figure 1:**
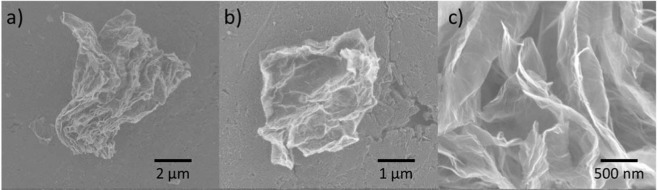
SEM photos of GF single particles.

The experiments were performed using the glass vessels with diameter 1 cm and length 3 cm. The weight of empty vessel was about 2 g. The vessels were filled with suspension of dispersed graphene foam particles (GF) of the sizes 1–10 µm in methanol (30 mg/cm^3^). The GF particles were obtained by using the method described in^19^. Morphology of the sample was analyzed using Jeol JSM-6610LVnx scanning electron microscope (SEM). The SEM imeges of GF particles are shown in Fig. 1. The EDX measurement of GF is presented in Supplementary Materials.

**Figure 2:**
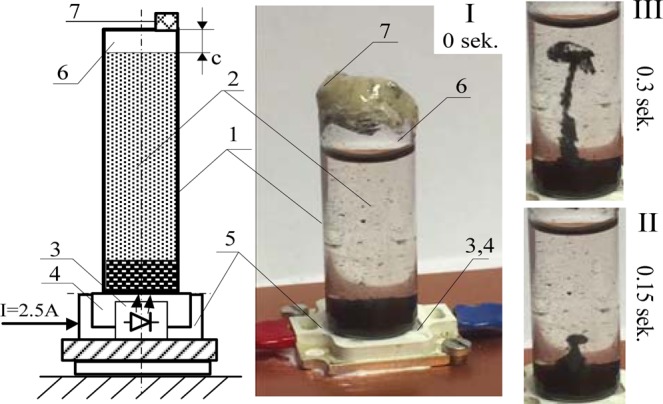
Sealed glass vessel (capsule) filled with GF particles and photos of GF movements in the liquid medium: 1 – cylindrical glass capsule, 2 – graphene suspension, 3 –LED matrix, 4 – silicone optical window, 5 – heat sink, 6 – air atmosphere, 7 – polymeric cup, II and III – photos of drifting GF particles in methanol solution upon irradiation after 0.15 s and 0.3 s.

The sealed vessel with the suspension of graphene foam (GF) particles dissolved and methanol was placed on LED module. The scheme of experimental setup was shown in Fig. 2. Preferably, the GF particles were sediment at the bottom of the dish. The following electrical parameters of GFP solution at the reset state were measured using two electrodes (as separate silver wires) inside the capsule - the static resistance was determined to be larger than 2 M Ohm, the static capacity less than 2 pF and the impedance was larger than 1MOhm at 100 KHz. All those parameters were strictly dependent on lighting intensity and varied during the tests due to the random, uncontrolled motions of individual GF particles.

The LED module (Epiled) was 9 element module emitting the infrared light 940 nm in active area 7.50 mm × 7.50 mm. Specification for the whole module was the following: total optical flux: up to 200 mW at square 10 × 10 mm^2^ at operation wavelength 940 nm, operating voltage for a single diode 1.4–1.6 V and max current 1000 mA for a single diode.

The experimental set-up for propulsion force measurement took into account the dynamic conditions of the propulsion effect. It consists from a capsule, LED module and ICP (integrated circuit piezoelectric) force sensor at two different configurations. In the first variant, the capsule starts from a flexible beam with stretched piezoelectric sensor and in the second one from a non-deformable plane. The second consists of the force sensor joined to the LED module mounted at the rigid steel plate. The main feature of propulsion effect of vessel is a rapid vertical launching against gravitation force. The analysis was performed using two methods: deflection of the beam fixed at one end and determination of reaction strength from the bottom of vessel.

## Results and Discussion

Figure 2 shows the scheme of experimental setup for observation of propulsion effect and its photograph illustrating behave of the capsule after switching on the illumination by LED module and initiating the effect. The first photo shows the vessel filled with suspension of methanol with GF particles sediment on bottom and placed on top of LED module. After switching on the light the GF particles shift quickly in direction along the light beam to the top of vessel. The observed propulsion of GF particles in solution is similar to the propulsion of GF particles in vacuum upon irradiation with laser diode^1^. It was noticed that the strength of propulsion was dependent of the irradiation power, amount of graphene particles and volume of air on the top of liquid. The propulsion did not occur if the vessel was fully filled to the top, without air space (see video in Supplementary Materials). A minimal threshold of propulsion effect (separation from module) defines the lowest level of necessary light emitter power. Even for low light irradiation the vessel was capable to jump perpendicularly to the surface along the irradiation direction.

The speed of capsule (vessel) was dependent on irradiation power and did not exceed 12 m/s in measurement conditions. The speed was measured by using high speed camera (Phanom Ametek V1611-Vision Research). The maximum height h_max_ of moving vessel may be expressed simply as h_max_ = v_0_^2^/2 g, where v_0_ is its velocity and g is the gravitational constant. Thus, the propulsion phenomenon makes possible to eject capsule up to several meters high, but the real height of the capsule was clipped by construction of testing stand. As described the maximal height of jumping vessel (reached even a few meters) was dependent on excitation light power. Useful exposure time does not cause a significant enhancement of temperature of the solution. The performed series of measurements by using a resistive electric heater have excluded a significant role of temperature growth since a propulsion effect did not occur. The propulsion force F_P_ was measured during of LED irradiation of capsule. It took few seconds (Δt) before the capsule jumped rapidly up. A mechanical response was measured in high sampling rate 1 MSPS (mega samples per second) at 16 bit resolution. For measurements of propulsion force two methods were applied: by using the piezo electric sensor converting small movements of beam to electrical signals interpreted as reaction force from the flexible substrate and by using the ICP sensor placed under the bottom of LED and capsule.

**Figure 3:**
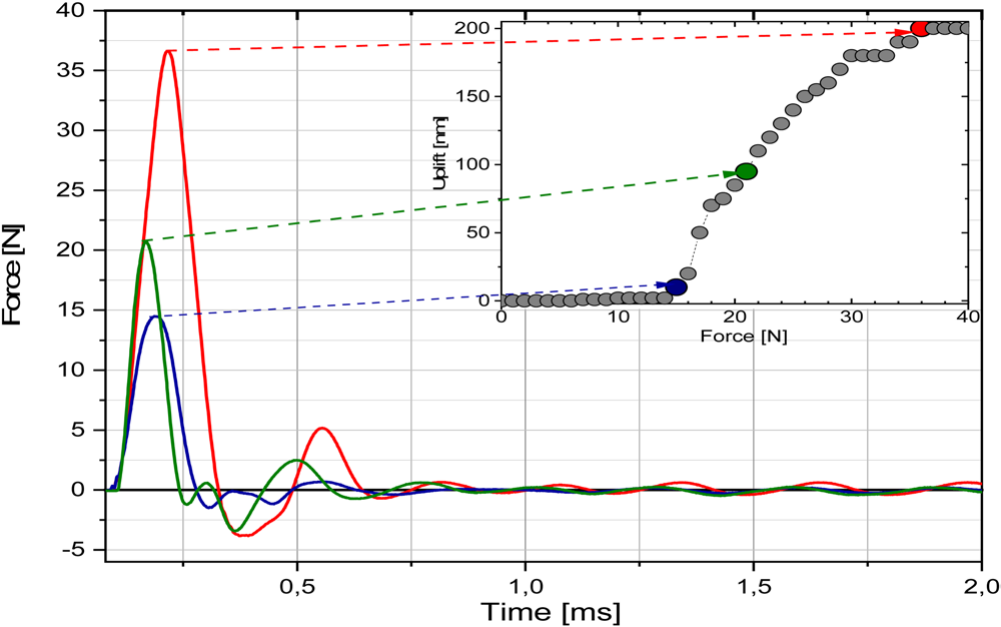
Time evolution of measured propulsion force F_P_ and linking of the measured propulsion forces F_P_ and the uplift height of jumping capsule (A) Correlation of the propulsion force F_P_ of capsule with GF solution measured by ICP sensor (B).

The temperature drift of the ICP sensor was observed due to heat transfer from LED module. The results were related with acquired a high speed pulse of vessel (or series of pulses) associated with the mechanical energy dissipated from the liquid and finally as reaction force in the sensor. The change of propulsion force is too fast for typical force measurement (even for ICP sensors), therefore the collected data were treated as a part of real propulsion force initiated from effect inside sealed vessel. The damping vibrations occurred due to rapid jump of the capsule from the beam. The schemes of measurement setups are shown in Fig. S1 (see Supplementary). The results of propulsion force F_P_ measurements are shown in Fig. 3. The examples of performed experiments are presented as movies in Supplementary. The glass vessel filled with suspension of methanol and 1 mg of GF particles was sealed with glue and subjected to irradiation of bottom with the light from LED module (as seen in Fig. 2). The mechanical wave propagated to LED matrix body from a sealed capsule can be detected by high speed and accurate force sensor. The examples of time evolution of measured propulsion forces at different applied electric powers are illustrated in Fig. 3.

**Figure 4:**
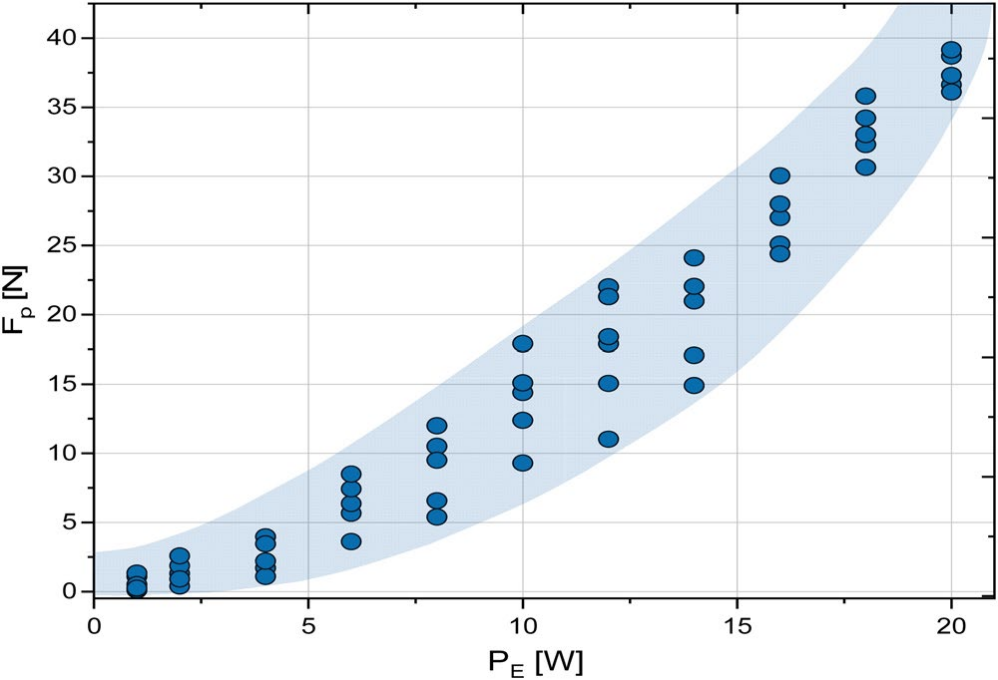
The dependence of propulsion force F_P_ on applied electric power P_E_ of LED module. The shaded region covers the experimental points of five different measurements fitting uncertainty.

The dependence of propulsion force F_P_ on applied power P_E_ of LED module is presented in Fig. 4.

One can see that the propulsion force F_P_ increased nonlinearly with applied electric power P_E_. A strong enhancement was observed for electric power enhancing 9 W. At this power the corresponding propulsion force F_P_ = 14 N and the capsule starts to jump. The high of jump reaches more than 200 mm for the propulsion force F_P_ = 40 N at the corresponding applied electric power of LED module P_E_ = 20.0 W.

### Discussion of propulsion mechanism

**Figure 5:**
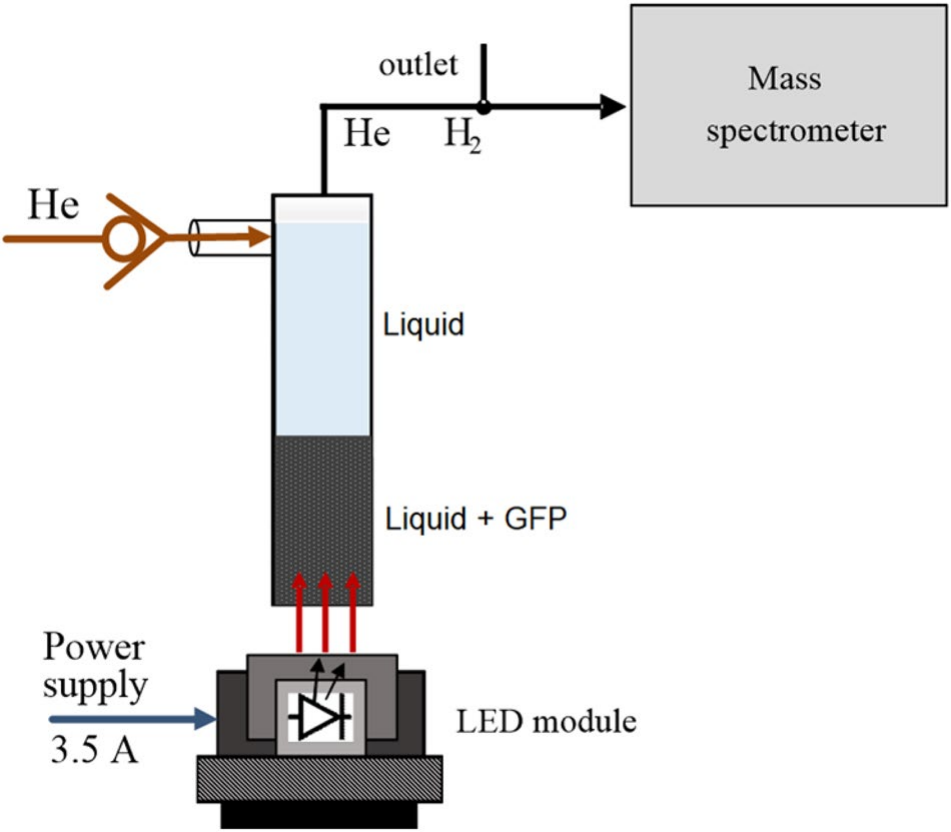
Scheme of measurement system of the hydrogen emission using a mass spectrometer of optically activated capsule with graphene particle suspension.

In course of experiments we have observed that GF particles in solution irradiated by CW LD light move horizontally to the top of vessel. The drift of GF particle occurs due to the emission of electrons from the graphene surface as was proposed by Zhang *et al*.^1^. The kinetic energy of moving electrons depends on the power of photon source. GF particles are hydrophobic in alcohol solution. The schematic illustration of interaction of light with GF particles is shown in Fig. 5. The hydrophobic GF particle is surrounded by local vacuum layer separating it from liquid. Due to interaction of photon *ћω* with graphene surface the free hot electrons e^−^ are ejected which kinetic energy is formulated by the photoelectric Einstein formula E_kin_ = *ћω* − φ, where φ is the work function of graphene (4.42 eV)^20^.

Graphene is characterized by zero-bandgap symmetric electronic structure at the Dirac point with the Fermi level between valence and conductive bands. Due to this the electrons behave as zero mass Dirac fermions with high velocity of 10^6^ m/s^11^. The hot electrons lead to dissociation of methanol and creation of negative hydrogen anion^21,22^.

$$2{{\rm{CH}}}_{3}{\rm{OH}}+{{\rm{e}}}^{-}\to 2{{\rm{CH}}}_{2}{{\rm{OH}}}^{+}+2{{\rm{H}}}^{-}$$

$$2{{\rm{H}}}^{-}\to {{\rm{H}}}_{2}+2{{\rm{e}}}^{-}$$

$${{\rm{H}}}_{2}+{{\rm{e}}}^{-}\to {{{\rm{H}}}_{2}}^{-}$$

In result there can appears the hydrogen molecules or the negative hydronic anions in methanol that may combine with cationic GF^+^ particle leading to adsorption of hydrogen molecule on a surface of graphene foam particle.

$${{\rm{GF}}}^{+}+{{\rm{H}}}_{2}^{-}\to {\rm{GF}}\in {{\rm{H}}}_{2}$$

Storing of hydrogen in alcohols is limited due to its low solubility. The negative hydronic H_2_^−^ ions may associate with positive GF particles (GF^+^ + e^−^) storing H_2_ molecules in pores. In results the effective density or porous GF particles decrease and they start to move up due to buoyancy. It is an alternative mechanism of transportation of GF particles. It is important to note that due to photoelectron emission the GF particle becomes positively charged GF^+^ and may capture two H^−^ negative hydrogen anions from solution which recombine in GF cavities following the reaction 2 H^−^ → H_2_↑ + 2e^−^ storing H_2_ molecules whereas free electrons e- remain in solvent. It means that the GF particle behaves as the hydrogen store in solvent. Due to absorption of hydrogen the effective density GF particles is lower and in result move to the top of solvent what is observed in our experiment (see Supplementary). The propulsion effect occurred due to explosion following combining hydrogen with oxygen 2H_2_ + O_2_ → 2H_2_O, when concentration of hydrogen exceeded 3,5%. This reaction of water synthesis is strongly exothermic 232 kJ/mol which rapidly releases of a much amount of energy in form of explosion.

### Mass spectrometry measurements of hydrogen emission

Is the mechanism of light induced propulsion associated with emission of hydrogen responsible for the explosive reaction H_2_ + O_2_? For this purpose we have performed the measurements of H_2_ emission by using mass spectrometer OmniStar QMS200 (Pfeiffer) (see the H_2_ calibration curve in Supplementary Materials). The block scheme of setup for measurement of H_2_ emission is presented in Fig. 5. The hydrogen emission was measured directly from the surface of suspension of GF particles in methanol using circulating open system with helium flow (10 ml/min).

**Figure 6:**
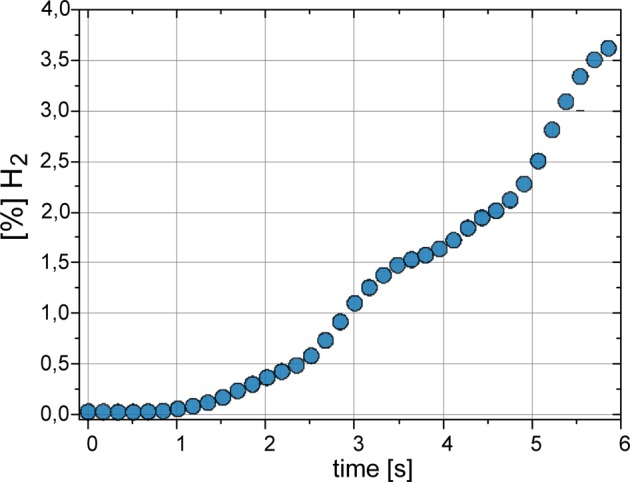
The time evolution of generated H_2_ gas concentration.

When we used high light power (1 W/cm^2^) generation of hydrogen started very fast and reached the level of hydrogen concentration in helium flow of about 3.5% after 6 s (see Fig. 6).

The successive steps of research should be combined with controlling of the hydrogen production using high power IR light irradiations.

## Summary

In the present work we have reported observation of extremely strong propulsion effect of closed glass vessel (capsule) filled with a suspension of methanol with dispersed GF particles upon illumination with infrared light emitting diode (LED) module. The propulsion effect was measured for the vessel subjected to the illumination in vertical movement. The propulsion force increased exponentially with illumination power and exceeded over 10^5^ times the gravity force of capsule. The mechanism of light induced propulsion was discussed in terms of multistep processes combined with an ejection of hot electrons from the graphene surface due to the photoelectron effect. In the second step the hot electrons interact with methanol molecule what leads to its fragmentation and production of H_2_ molecules. In third step the H_2_ molecule interacts with electron e- leading to its recombination to negatively charged hydronic H_2_^−^ ion. In next step hydronic ions are adsorbed by positively charged (cathode) GF particles recombining to neutral H_2_ molecules. Since GF has a huge effective surface area it becomes a reservoir (adsorber) for hydrogen. The GF particles with adsorbed hydrogen are characterized by lowered effective density and are transported to the gas phase located at the top of the vessel. When the concentration of hydrogen enhances to about 4% in the air there is initiated the high energetic explosive reaction leading to the fast vertical self-propulsion of the capsule. The propulsion strength should be easily controlled by tuning of supplied light power. The further studies of light-induced propulsion of vessels filled with different liquids and by using different excitation sources also including lasers are in progress.

## Supplementary information

Supplementary materials.

Propulsion of graphene foam.

Force_measurement_setup.

No jump of full filled capsule.
