# Supplementary material for: Direct light-induced propulsion of vessels filled with a suspension of graphene particles and methanol
Source: Sci Rep. 2020 Feb 10;10:2222. doi: 10.1038/s41598-020-59123-y (PMC7010679; doi:10.1038/s41598-020-59123-y)
Supplement: Supplementary file 1 — Supplementary materials. [file 41598_2020_59123_MOESM1_ESM.docx]

Supplementary Materials for

**Direct light-induced propulsion of vessels filled**

**with a suspension of graphene particles and methanol**

W. Strek, P. Wiewiorski, W. Mista, R. Tomala^*^

Institute of Low Temperature and Structure Research,

Polish Academy of Science, Wroclaw, Poland

*Corresponding author: [r.tomala@intibs.pl](mailto:r.tomala@intibs.pl)

**SEM-EDX analysis**

Scanning electron microscopy (SEM) images and energy dispersive X-ray (EDX) analysis were taken on a field emission scanning electron microscope (FE-SEM, FEI NovaNanoSEM 230 equipped with an EDS analyzer EDAX Genesis XM4).

Films for SEM and EDX analysis were prepared by placing the sample suspension on a silicon wafer sample holder and then drying at room temperature. The EDX analyses were performed from the large area (250μm × 200μm) of samples.

GF had an oxygen content of 7.66 atom%, as shown in Figure S1, the atomic ratio of carbon to oxygen was 12.0.

Fig S1. Typical EDX spectrum at 5keV electron beam referred to GF sample deposited on silica wafer (* Si Kα support = 1.74keV)

Fig S2. The Mass spectrometer calibration curve for H_2_.

The schemas of the experimental methods are presented in Fig. S1. Only “mode 1” and “mode 2” are described in the paper.

| A) | B) | C) |
| --- | --- | --- |
|  |  |  |

Fig. S3. Methods for determining the mechanical response during the initiation of the propulsion: beam deflecting – mode 1 A), reaction strength – mode 2 B), mode 3 as explosion state.

**Measurement set-up for evaluation of propulsion effect**

The scheme of the measurement set-up is shown on the Fig. S4 mm in HD resolution.

Fig. S4. Acquisition system dedicated for high speed propulsion detection: LED – light source, ICP – force sensor, RPS- regulated power supply, SWT – switch, TMP – temperature measurement, PCB – ICP sensor conditioner, CAM – 240fps camera, ACQ – main processing system with software.

Main parts of the measurement set-up.

LED – 3x3 matrix power LED 940nm, single element - 45mils,

ACQ – Acquitek CM-2222 – 1MHz 16bit resolution ADC (Analog Digital Converter),

PZT – DuraAct piezopatch

PCB – PCB ICP force sensor 11.5mV/N – used in the Mode 2,

TMP – PT100 sensor with Pico Technology PT104 logger system,

CAM – Cisco camera,

SWT – Mosfet power switch,

RPS – Regulated power supplies as current source 0-3500mA,

Software – Dasylab 2016.

The following parameters were recorded:

1. mechanical response from sensor:

- Mode 1 – voltage from piezopatch,
- Mode 2 – force from ICP sensor,

1. temperature of LED module for alarm conditions,
2. LED current,

Mechanical response was measured in high sampling rate 1MSPS (Mega Sample Per Second) at 16bit resolution. In addition to the parameters mentioned, acoustic events were analyzed.

The schemes of the propulsion force measurements are presented in Fig. S5. The measured value of the propulsion force in the Mode 1 stand was very small. The damping vibrations were occurred due to rapid loss of the capsule from the beam. The propulsion of sealed vessel is a very fast phenomenon, requiring a different measurement technique (Mode 2)

| A |  | B |  |
| --- | --- | --- | --- |

Fig. S5. Scheme of measurement setup of light induced propulsion of sealed glassy vessel filled with solution of graphene suspension: A - start from beam (mode 1), B - start from non-deformable plane (mode 2) - 1 – cylindrical glass vessel, 2 – graphene solution, 3 – power LED matrix, 4 – silicone optical window, 5 – heat sink, 6 – gas bubble, 7 – valve (hydrogen collection system), 8 – piezoelectric accelerator sensor (ICP PCB), 9 – rigid plate.

The following parameters were recorded: the mechanical response from accelerator sensor (voltage from piezopatch) and the force from ICP sensor, and simultaneously the temperature of LED module and the LED current. An ICP quartz piezoelectric force transducer is a sensor that generates an electrical output proportional to applied impact force. ICP sensors are designed to measure vibration and shock forces for a wide variety of ballistic applications. They are simple to use and accurate over a wide frequency range up to 200 KHz which makes them the recommended choice for identification of the propulsion effect.

The propulsion force F_P_ was measured during of the LED irradiation of capsule. The irradiation of the capsule took a few seconds (Δt) before the capsule jumped rapidly up. A mechanical response was measured in high sampling rate 1MSPS (Mega Sample Per Second) at 16bit resolution. The propulsion force was measured by two methods: by using the piezopatch sensor converting small movements of beam to electrical signals interpreted as reaction force from the flexible substrate and by using the ICP sensor placed under the bottom of LED and capsule. The temperature drift (D_V_) of the ICP sensor was observed due to heat transfer from LED module. The results are related with acquired a high speed pulse F_P_ (or series of pulses) associated with the mechanical energy dissipated from the liquid and finally as reaction force in the force sensor. The propulsion force is too fast for typical force measurement (even for ICP sensors), therefore the collected data were treated as a part of real propulsion force initiated from effect inside sealed vessel.

The schemes of measurement setups for propulsion force are shown in Fig. S1 (see Supplementary).

| A) |  |
| --- | --- |
| B) |  |

Fig. S6. Experimental methods of propulsion force F_P_ measurement of ejected capsule containing GF solution from the flexible beam. The time evolution of propulsion force F_P_ affecting on vessel with GF solution irradiated with LED measured by ICP sensor.

1. **Propulsion from flexible beam fixed at one end**

| A) |
| --- |
|  |
| B) |
|  |

Fig. S7. Experimental setup for determining beam force reaction F_P_ measurement during propulsion effect A), small beam reaction F_P_ effect t during propulsion of the capsule containing GFsolution B).

1. **Propulsion from rigid plane**

Fig. S8. Investigation methodology of light induced propulsion of capsules with GF dissolved in solution
